# Infectious diseases as ‘a colourful universe of possibilities’

**DOI:** 10.4102/sajid.v37i2.407

**Published:** 2022-02-14

**Authors:** Nelesh P. Govender

**Affiliations:** 1 Centre for Healthcare-Associated Infections, Antimicrobial Resistance and Mycoses, National Institute for Communicable Diseases, a Division of the NHLS, Johannesburg, South Africa; 2 School of Pathology, Faculty of Health Sciences, University of the Witwatersrand, Johannesburg, South Africa

[*T*]rainees choosing to enter [*infectious diseases*] encounter … a blank canvas of exciting possibilities from which they can compose career paths in directions of their choice, using a vibrant palate [*that*] ranges from microbiology, molecular biology, pharmacology, host defence, clinical science, and patient care to epidemiology, public health, behavioural science, health disparities, implementation science, global health, medical education, and health economics, to name just a few. The canvas is their clinical and scientific playground, where they mix and merge a wide variety of disciplines in a style that fits their interests, needs and desire to make a difference. It is fluid, dynamic and tremendously exciting.^1^ (p. 581)

In the aftermath of the second coronavirus disease 2019 (COVID-19) wave in South Africa, during which members of the Federation of Infectious Diseases Societies of Southern Africa (FIDSSA) had been drawn into every aspect of the response from front-line clinical care to epidemic control and cutting-edge vaccinology and science research, I proposed the Wisdom series to the FIDSSA Council. The pandemic was an acute reminder that healthcare professionals trained in infectious diseases are essential to the integrity of South Africa’s health system. Despite this, a very few people choose to pursue a medical, nursing or scientific career focussed on infectious diseases. Simultaneously, many specialists in the field have chosen to leave, seeking greener pastures. Even before the pandemic, we had recognised that the infectious diseases medical speciality was in crisis.^2^ I considered my own meandering pathway into a neglected field at the intersection of laboratory medicine, infectious diseases and public health, and remembered that things were not always clear to me. Recently qualified professionals deciding on an area of speciality do not know what to expect from a career in infectious diseases. The ‘colourful universe of possibilities’ within an infectious diseases career can appear to be murky and disconcerting to fresh eyes. Even a newly qualified specialist can take a long time to choose their own adventure, and of course, even a carefully planned career path may change unexpectedly with chance events and opportunities. I have been incredibly fortunate in my own career to find willing and wise mentors in Professors Lucille Blumberg, John Frean, Shabir Madhi, Graeme Meintjes and Tom Harrison. I have also had the privilege of mentoring talented young scientists and clinicians.

The Wisdom series is targeted at junior and mid-career FIDSSA members. The aim of this series was to outline the diversity of career trajectories in infectious diseases in South Africa and to provide a permanent library of mentor and mentee resources for its members. Using a mixture of written contributions and pre-recorded interviews, we invited respected people in the local infectious diseases community to reflect on their personal career trajectories, the impact of their work, the mistakes they had made along the way, and the advice they would provide to FIDSSA members for happy and fulfilling careers. We asked FIDSSA members to nominate people working in any of three main tracks – academic, clinical and public health – and aimed to include a good mix of contributors in the middle of their careers, as well as experienced wise voices, reflecting the broader membership.

The contributions have exceeded my expectations. What follows in these pages and in the recorded interviews online are brutally honest reflections and practical lessons from the interviewed contributors. I have included some quotations below from three of the interviewed contributors. The full recordings of the interviews will be available through the FIDSSA website.

*Professor Shaheen Mehtar*, infection control:

‘Infectious diseases is such an exciting speciality where things just keep changing all the time. You never get bored. Because you are working with a parallel universe of live microbes and you are trying to outwit each other all the time’.

‘You want to support your teams in such a way that they trust you with the information’.

*Professor Koleka Mlisana*, clinical microbiology – ‘Set your mind to doing things well’; ‘[f]or you to be passionate about what you do, you must train yourself to excel’.

*Professor Lucille Blumberg*, infectious diseases:

‘Acknowledge your small victories. They will give you energy to persist’. ‘[f]ind your space and see what you can do’; ‘[y]ou have to grab and grow your opportunities’.

[*T*]here are things that you cannot change but look for things that you can. Look for ways of doing it. Be encouraged when things do work. There are days when you want to give up. Remember the ones where you do make a difference’.

I would like to thank and acknowledge all our contributors for the time taken to prepare their pieces, Drs Kirti Ranchod and Anastasia Koch for their help in compiling the Wisdom series, Ms Lea Lourens and Dr Gary Reubenson for editorial assistance and our sponsors, Merck Sharp & Dohme Corp. (MSD; a subsidiary of Merck & Co., Inc.) and bioMerieux, for generously providing educational grants to FIDSSA to support the Wisdom series.
